# Measuring Coverage in MNCH: Indicators for Global Tracking of Newborn Care

**DOI:** 10.1371/journal.pmed.1001415

**Published:** 2013-05-07

**Authors:** Allisyn C. Moran, Kate Kerber, Deborah Sitrin, Tanya Guenther, Claudia S. Morrissey, Holly Newby, Joy Fishel, P. Stan Yoder, Zelee Hill, Joy E. Lawn

**Affiliations:** 1 Save the Children, Washington, District of Columbia, United States of America; 2 Save the Children, Cape Town, South Africa; 3 University of Western Cape, Belleville, South Africa; 4 Statistics and Monitoring Section, Division of Policy and Strategy, United Nations Children's Fund, New York, New York, United States of America; 5 ICF International, Calverton, Maryland, United States of America; 6 Institute of Child Health, London, United Kingdom; Independent consultant: Health statistics and policy

## Abstract

Neonatal mortality accounts for 43% of under-five mortality. Consequently, improving newborn survival is a global priority. However, although there is increasing consensus on the packages and specific interventions that need to be scaled up to reduce neonatal mortality, there is a lack of clarity on the indicators needed to measure progress. In 2008, in an effort to improve newborn survival, the Newborn Indicators Technical Working Group (TWG) was convened by the Saving Newborn Lives program at Save the Children to provide a forum to develop the indicators and standard measurement tools that are needed to measure coverage of key newborn interventions. The TWG, which included evaluation and measurement experts, researchers, individuals from United Nations agencies and non-governmental organizations, and donors, prioritized improved consistency of measurement of postnatal care for women and newborns and of immediate care behaviors and practices for newborns. In addition, the TWG promoted increased data availability through inclusion of additional questions in nationally representative surveys, such as the United States Agency for International Development–supported Demographic and Health Surveys and the United Nations Children's Fund–supported Multiple Indicator Cluster Surveys. Several studies have been undertaken that have informed revisions of indicators and survey tools, and global postnatal care coverage indicators have been finalized. Consensus has been achieved on three additional indicators for care of the newborn after birth (drying, delayed bathing, and cutting the cord with a clean instrument), and on testing two further indicators (immediate skin-to-skin care and applications to the umbilical cord). Finally, important measurement gaps have been identified regarding coverage data for evidence-based interventions, such as Kangaroo Mother Care and care seeking for newborn infection.

## Abstract 2

In a *PLOS Medicine* Review, Allisyn Moran and colleagues introduce the work of the Newborn Indicators Technical Working Group (TWG), which was convened by the Save the Children's Saving Newborn Lives program in 2008, and describe the indicators and survey questions agreed upon by the TWG to measure coverage of care in the immediate newborn period.

*This paper is part of the* PLOS Medicine *“Measuring Coverage in MNCH" Collection.*

## Introduction

Neonatal mortality accounts for 43% of under-five mortality [1] and is becoming a political priority at both global and country levels [2],[3]. Ministries of Health, partners, and donors are increasingly focused on implementing packages and specific interventions aimed at improving newborn health and survival [4]. To assess changes and improvements, accurate and accessible data on coverage of evidence-based interventions are essential. However, few of the highest impact interventions for newborn care are systematically measured [4].

Given the relatively recent focus on scale-up of newborn care, and the fact that many of the interventions are linked to packages of care for women or children, there has been limited agreement on indicators to monitor and evaluate newborn care. To address this gap, Save the Children's Saving Newborn Lives program convened the interagency Newborn Indicators Technical Working Group (TWG) in 2008 focused on improving the capture and measurement of newborn care. This group of about 20 individuals includes people from United Nations agencies, nongovernmental organizations and national institutes of health, researchers and academics, evaluation and measurement experts, donors, and other stakeholders. The group aims to reach consensus on key indicators, including definitions and standard measurement tools. It also promotes inclusion of agreed-upon indicators in nationally representative household surveys such as the United Nations Children's Fund–supported Multiple Indicator Cluster Surveys (MICS) and the United States Agency for International Development–supported Demographic and Health Surveys (DHS) [5]. In addition, the TWG disseminates standard indicators and tools for inclusion in specialized data collection efforts at subnational levels. The main TWG group meets twice per year, with subgroups working on particular areas meeting more frequently.

The objective of this article, which is part of the *PLOS Medicine* “Measuring Coverage in MNCH" Collection, is to describe the process of developing and achieving consensus on indicators to monitor and evaluate coverage of evidence-based interventions for newborns. The TWG's initial focus was on three areas of measurement: postnatal care, immediate care behaviors and practices for newborns, and health facility assessments. We will focus on achievements in the first two areas, provide recommendations for indicators and tools, and identify areas for future research efforts.

## Measurement of Coverage of Postnatal Care

Around three-quarters of neonatal and maternal deaths occur in the first week of life, with up to half in the first 24 hours [6]. Postnatal care to provide counseling and to identify and treat complications after birth for both the mother and her baby was identified by the World Health Organization (WHO) as a critical care package at an expert review meeting in 1997 and has been included in its guidelines for pregnancy, childbirth, postnatal, and newborn care since 2003 [7]. Research has demonstrated that a postnatal care home visit from a trained provider within two days of delivery can result in 30% to 60% reduction in neonatal mortality [8]–[10]. The evidence is less clear for maternal outcomes, mostly due to challenges with measuring maternal mortality. Nevertheless, the 2003 WHO guidelines integrate care for the woman and for her baby from shortly after childbirth until six weeks after birth, as such care is often provided at the same time by the same provider. In 2007, postnatal care was redefined as being for both woman and newborn in the context of the continuum of care [11]. Further, the Countdown to 2015: Maternal, Newborn and Child Survival initiative defines postnatal care as integrated care for women and their newborns [12],[13].

Given the importance of the postnatal period and the clear evidence of the effectiveness of early postnatal care for newborns, an indicator that measures a contact with a health worker to provide counseling and to assess health within the first two days after birth is critical for global monitoring. Countdown to 2015, which was established in 2005, includes early postnatal care for women and newborns in its initial list of priority indicators [13]. More recently the Commission on Information and Accountability for Women's and Children's Health prioritized early postnatal care as one of seven selected coverage indicators to measure progress for maternal, newborn, and child health [14]. Although there is consensus on the importance of care provided during this time period, there are many challenges associated with measuring coverage in large-scale household surveys (Table 1). Each of these challenges was discussed during TWG meetings, research and secondary analyses were conducted, and consensus on an indicator for postnatal care within two days of birth regardless of delivery location was achieved (Table 2). Following TWG deliberations, this indicator has been tested by MICS, and data collection has been harmonized across DHS and MICS surveys [15].

**Table 1:** Postnatal care indicator: measurement issues and advances.

Topic	Issue(s)	What Has Been Accomplished	What Needs to Be Done
Recall and validity	• Uncertainty about mother's knowledge about what happened to baby after birth, especially for facility births• Lack of recall of past births up to five years prior to survey• Potential misunderstanding of survey questions on postnatal care	• Formative research indicates that women have a good idea what happens to their baby regardless of where they deliver• DHS and MICS questionnaires revised to include postnatal care for all newborns, regardless of place of birth• Formative research indicates that women have difficulty understanding the term “postnatal care"• DHS and MICS questionnaires revised to include an introductory statement for postnatal care questions• Standard tables in DHS and MICS updated to include postnatal care coverage for newborns	• Review data from new DHS and MICS questionnaires and revise tools as needed
Timing	• Lack of criteria to distinguish between intrapartum and postnatal care, e.g., should all contacts from birth count, or is postnatal care valid only if it takes place after the intrapartum period• Potential overestimation of true postnatal contacts	• Detailed postnatal care module developed and tested for MICS• Distinct measurement of the first pre-discharge and first post-discharge contact in MICS	• WHO recommendation to define postnatal contact (e.g., a cutoff of one hour after birth)• Formative research to differentiate intrapartum and postnatal care• Implementation research on facility pre-discharge checklist, etc.
Number of visits	• Only the first postnatal contact captured in DHS and MICS surveys, and may be an intrapartum contact but no further question asked• Lack of ability to capture pre-discharge postnatal care more accurately• Lack of data on home or clinic postnatal contacts after facility births	• Detailed postnatal care module developed and tested for MICS• Distinct measurement of the first pre-discharge and first post-discharge contact in MICS• No information captured on subsequent postnatal contacts	• Capture additional visits in optional module or specialized surveys
Content	• Data not currently collected in national surveys	• Formative research indicates women could recall specific actions for newborns during postnatal care (such as use of equipment, undressing baby, giving advice)• Consensus on five measureable signal functions for postnatal care for newborns	• Work with maternal health community on signal functions for postnatal care for women• WHO meeting to define postnatal care interventions for women and newborns• Test household survey module for content of postnatal care for newborns and women

**Table 2:** Global indicators for postnatal care coverage, 2010.

Category	Indicator	Numerator	Denominator
Postnatal care for women	Percent of women who received postnatal care within two days after last delivery	Number of women who received postnatal care within two days after last delivery	Number of women with a live birth in the last two years
Postnatal care for newborns	Percent of newborns who received postnatal care within two days after delivery	Number of newborns who received postnatal care within two days after delivery	Number of women with a live birth in the last two years

### Measurement Challenges and Recommendations

Similar to other long-standing, global indicators such as antenatal care and skilled attendant at delivery that focus on contact with the health system, the measurement focus for postnatal care is a contact with a health care provider. This contact provides the platform for delivering lifesaving interventions, but measurement of contact does not provide any indication about the content or quality of care. Measurement of the first postnatal care contact provides a complementary indicator to antenatal and delivery care, and extends the ability to monitor contacts across the continuum of care from pregnancy through childbirth and the postnatal period [16].

Postnatal care includes a specific contact for both the woman and her newborn. It is distinct from intrapartum care, which is provided by the delivery attendant to the woman and her child around the time of birth. Postnatal care focuses on prevention of complications through counseling on important health messages, assessment of the woman and newborn, and referral for complications, if identified. Although a postnatal contact should contain elements of care for both women and newborns, this does not always happen in programs. As a result, for the time being it is necessary to measure postnatal care for the woman and newborn separately to ensure that an inclusive contact has taken place [16].

DHS surveys collect data on postnatal care for women who gave birth either at home or in a health facility, but, historically, most surveys have collected postnatal care data for newborns only for those born at home. This distinction is based on the untested assumption that women who give birth in facilities will have difficulty knowing whether their newborn received postnatal care. Based on recent qualitative work in Bangladesh and Malawi [17] and in Ghana (Hill et al., unpublished report, 2010, Institute for Child Health, University of London), in which women with facility deliveries were able to answer questions about postnatal care contact for their newborns (Box 1), the standard indicator has now been modified to include all births regardless of place of delivery (Tables 1 and 2). The reference period has also been revised to include births occurring within two years preceding the survey (as opposed to five years), which is long enough to provide enough cases for analysis, but not so long that it may hamper recall. To date, 25 countries have collected data on postnatal care coverage for women, and four have collected data on postnatal care coverage for newborns using this definition [13].

**Box 1. Qualitative Research Studies to Assess Postnatal Care and Immediate Care Behaviors and Practices for Newborns**

The TWG supported two qualitative studies to assess postnatal care and immediate care behaviors and practices for newborns—one in rural Ghana in 2010 (Hill et al., unpublished report, 2010, Institute for Child Health, University of London) and one in Bangladesh and Malawi in 2009 [17]. Both studies used birth narratives to describe the birth process and immediate newborn care. The narrative approach is used to understand what is salient by probing specific areas, an approach that is more appropriate for low-income developing country settings than cognitive testing. In Ghana, the narratives were supplemented with focus groups to assess the terminology used to describe postnatal care and interviews with health workers. In Bangladesh and Malawi, the narratives were followed by a structured questionnaire on specific care behaviors and practices, including the timing of events. Women with a birth within the past year were interviewed in both studies, stratified by place of birth and time since delivery.

In Ghana (40 birth narratives, four focus groups, ten health worker interviews)

- Questions on postnatal care were not easily understood by women.
- Few women gave spontaneous answers about the postnatal care that they or their newborn had received.
- Probing phrases about specific activities that could take place during postnatal care were essential.
- Questions had to be carefully phrased to allow for local terminologies and perceptions.
- Mothers were most likely to report an observed specific action during postnatal care such as the use of equipment or undressing the baby.
- Being tired or sick after delivery, time since delivery, number of visits, outcome of the visits, and where the newborn was cared for after facility-based birth influenced recall of postnatal care.

In Bangladesh and Malawi (84 and 80 narratives/questionnaires, respectively)

- The narratives indicated that women can recall the main birth events (their labor pains, the delivery of the placenta, cord care, and newborn care) regardless of place of delivery.
- There was limited difference in recall between home and facility births.
- The majority of women gave nonnumeric answers to questions about timing of events.
- When asked how long after delivery their checkup occurred, answers ranged from minutes to days.
- Many women did not understand what was meant by a postnatal care contact defined as a “health checkup," or “a check on your health" after the baby was born unless study teams defined the term. In Malawi, interviewers provided examples of postnatal care content; in Bangladesh, interviewers described what was meant by health in general.

All health care providers are included in the current definition of the global postnatal care indicator (including traditional birth attendants and community health workers), regardless of the skill level of the provider. This differs from the current global standards for at least one antenatal care visit and skilled attendance at delivery, which are based on contact with a skilled provider. Although information on where postnatal care took place (e.g., at home or at a health facility) is of programmatic interest, it is not reported on separately in the global indicator. Postnatal care can be effectively provided by a variety of health workers, ranging from community health workers to skilled providers, and may differ for the woman and newborn, as well as the place of care [10]. The global indicator therefore includes postnatal care for mother and newborn by any provider, but standard tables in both DHS and MICS reports disaggregate coverage by type of provider and place of birth.

Another critical challenge facing the measurement of postnatal care contact is the element of timing. The postnatal period refers to the first 42 days following delivery for both the woman and the newborn. Recent WHO and UNICEF guidelines recommend a postnatal care visit for mother and newborn on day 1, day 3, and day 7 after birth, with continuing contacts throughout the first six weeks of life [10]. Evidence from Bangladesh has demonstrated that a postnatal visit to the newborn in the first 48 hours can significantly reduce mortality, whereas first postnatal contacts after that time were not associated with reduced mortality [8]. Postnatal care within two days of delivery aligns well with measurement in household surveys, given that there is potential misclassification in recall on the day of delivery and the day after delivery.

The global postnatal care indicators recommended by the TWG are useful for standard measurement across large-scale household surveys to monitor progress toward achieving coverage along the continuum of care. The global indicators alone, however, are not sufficient for programmatic needs. Programs that focus on improving maternal and newborn survival and health should include additional questions on other key elements of postnatal care in the surveys used to monitor progress, including timing of each postnatal care contact, location and provider of postnatal care, and, ideally, content of care. The TWG has agreed on five signal functions to assess the content of postnatal care for the newborn. These functions are part of the recommended postnatal care package and are considered feasible for reporting by women in surveys based on qualitative work in Ghana (Box 1) and on analysis of Saving Newborn Lives household surveys (Box 2). The signal functions are (1) checking the newborn's umbilical cord, (2) assessing the newborn's temperature, (3) observing/counseling on breastfeeding, (4) counseling on newborn danger signs, and (5) weighing the baby, if appropriate for a given country. The TWG suggests including these signal functions in an optional module in nationally representative surveys, and in smaller subnational surveys where there is interest in exploring these areas, although consideration must be given to the length of questionnaires as well as data quality. Finally, there have also been preliminary discussions with the TWG and external experts on signal functions for postnatal care for the woman, but more discussion is needed with the wider community.

**Box 2. Survey Questions on Newborn Care Practices: Summary Results and Recommendations**

Save the Children's Saving Newborn Lives program conducted household surveys in five countries in Asia, Africa, and Latin America, maintaining standardized measurement of key indicators (drying, delayed bathing, and cutting the cord with a clean instrument) across countries as much as possible. Multiple questions were often used to measure the same indicator, which provided the opportunity to test different question formulations. In each country, cross-sectional household surveys with pre/post design using community cluster sampling were conducted among women with a live birth in the 12 months prior to the survey (nine months for Bangladesh). Countries surveyed included Malawi (2011, *n = *900), Bangladesh (2010, *n = *794), Nepal (2011, *n = *630), Viet Nam (2011, *n = *1,050), and Indonesia (2011, *n = *400). Target sample sizes were calculated based on expected changes in key indicators, ranging from around 600 women in Nepal to 900 women in Malawi.

- For drying and delayed bathing there were low rates of “don't know" or missing data responses (<10%).
- For cord care, there were differences between women with home and with facility births.
- For home births, women were more able to report the type of instrument used to cut the cord, compared with facility births.
- Rates of “don't know" and missing data among women with facility births were around 30% in Malawi, compared with less than 5% among women with home births.

Based on these findings, the TWG recommends the questions shown in Figure 3 for use in household surveys.

### Remaining Research Gaps

**Figure 1:**
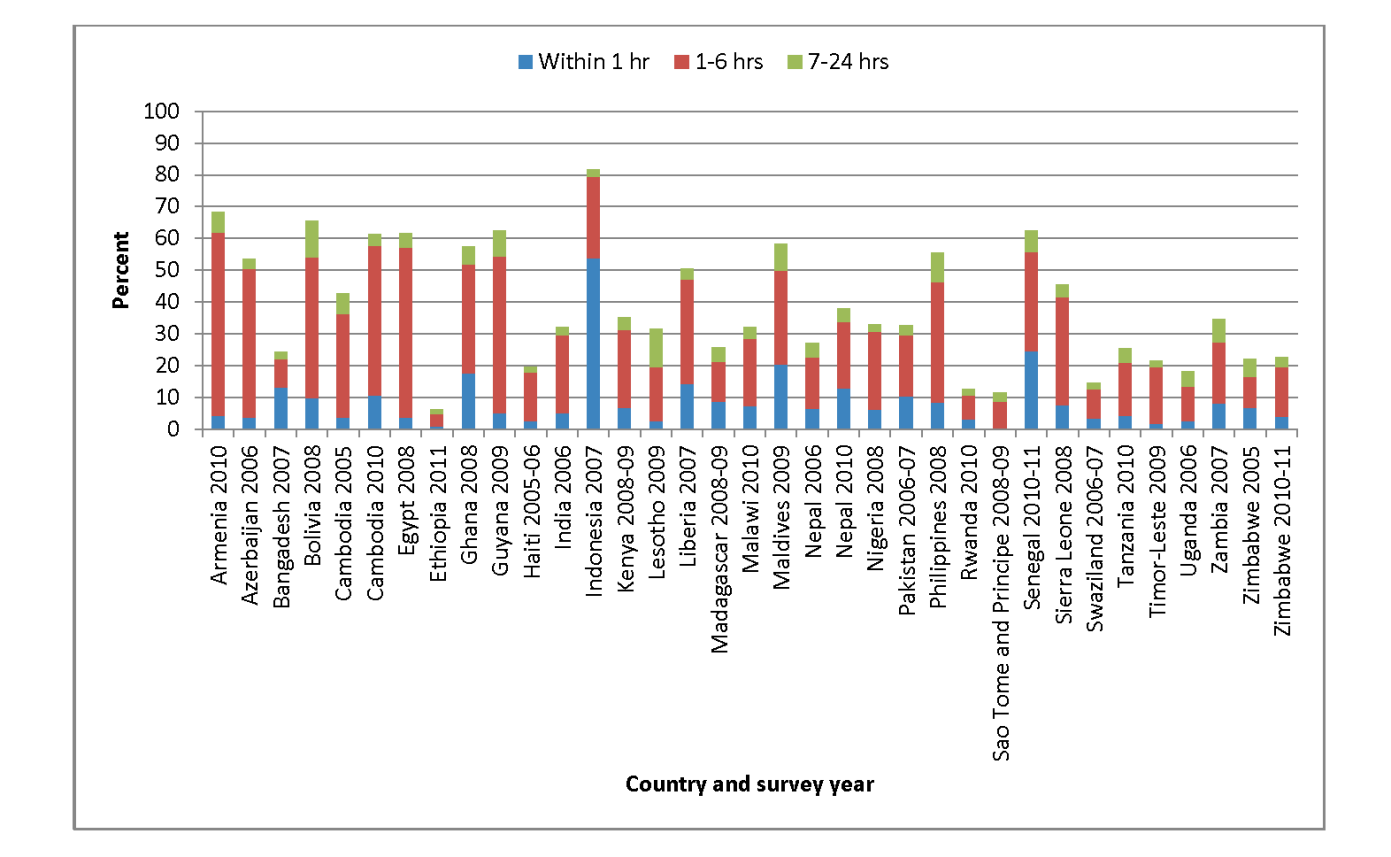
Proportion of women who received postnatal care within two days of delivery by time of first visit, DHS survey data 2005–2011 [**18**].

The first important area where additional research is needed is the timing of postnatal care. The end of the postnatal period is relatively well defined, although there is global discussion about extension of the postnatal period for the woman beyond the currently accepted 42 days after delivery. However, there is much less consensus around when the intrapartum period ends and the postnatal period begins. For example, maternal health experts typically define the intrapartum period as ending after the delivery of the placenta, whereas newborn health experts typically set one hour after birth as the end of this period. As there are few countries with postnatal care coverage data for newborns among all births, to get an idea of how much reported postnatal care might actually be intrapartum care, we used recent DHS data from 35 countries to assess postnatal care coverage for women [18]. Our analysis indicates that 35% of postnatal care contacts occur within six hours of birth (Figure 1). Thus, many of these contacts, though important, could be part of routine intrapartum care and not what most programs would consider distinct postnatal care contacts.

WHO conducted a meeting in September 2012 to review postnatal care guidelines, including recommendations for timing of discharge after facility delivery, the number and timing of postnatal care contacts, and the content of those contacts. Part of the discussion was focused on defining the “postnatal period" and differentiating postnatal care from intrapartum care for both women and newborns (results are forthcoming), which may assist in resolving some of the measurement challenges referred to above. However, it is important to consider that even if consensus on a particular cutoff were reached, it would be difficult to operationalize this definition in large-scale household surveys. First, women's recall of a contact occurring before, at, or after four, five, or six hours after birth may not be reliable, especially two years after the birth. Second, standard data collection currently includes only the first postnatal care contact, so if additional contacts take place, women do not have the opportunity to report them in the survey, which may result in an underestimation of the overall coverage.

Another important research gap concerns the providers of postnatal care. Currently, as discussed earlier, the global indicator for postnatal care includes all health care providers regardless of their skill level. More research is needed to determine whether traditional birth attendants should be removed from the indicator definition since they are rarely trained in postnatal care programs. Although it can be difficult to identify skilled health personnel reliably, other indicators such as antenatal care and skilled birth attendance also rely on this identification [16].

**Figure 2:**
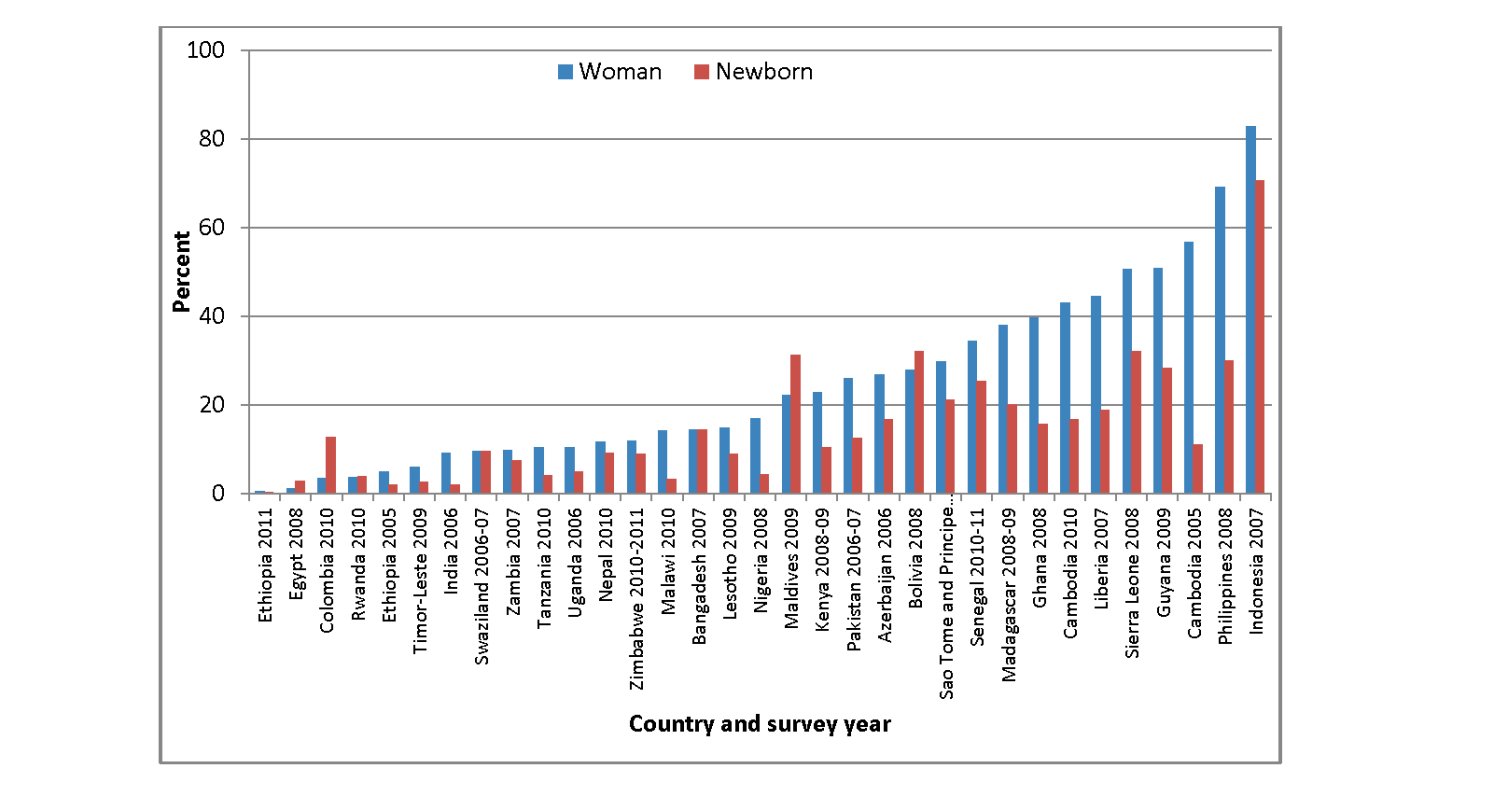
Proportion of home births for which women and babies received postnatal care within two days of delivery, DHS survey data 2005–2011 [**18**].

Another issue for future consideration is the feasibility of combining the measurement of postnatal care contacts for women and newborns. Ideally, these contacts should take place at the same time and include elements for both the woman and her newborn. However, based on our analysis of DHS data from 33 countries between 2006 and 2011 [18], there is great variability in terms of postnatal care coverage for women and newborns (Figure 2). In Bangladesh and Zambia, postnatal care coverage for home births is similar for women and their newborns (14% for both women and newborns in Bangladesh; 10% for women and 8% for newborns in Zambia). However, in Ghana, 40% of women who gave birth at home reported receiving postnatal care for themselves, but only 16% reported that their newborns received care. Similarly, in Cambodia, 43% of women who gave birth at home reported receiving postnatal care for themselves, but only 17% reported postnatal care for their newborns. This discrepancy, which may be due to measurement error, to misunderstanding the questions, or to actual differences in coverage, requires further exploration, especially as more comparable data (for all births regardless of place of delivery) become available in the next several years.

## Measurement of Coverage of Immediate Care Behaviors and Practices for Newborns

The second area of measurement that the TWG specifically concentrated on during its deliberations is care of the newborn immediately after birth, which includes a number of functions. Three of these functions can be provided at both community and facility levels: (1) thermal care (drying, skin-to-skin care, and delayed bathing), (2) hygienic and clean skin and cord care (clean cord cutting and dry cord care; hand washing prior to delivery), and (3) breastfeeding (immediate initiation; not discarding colostrum; no foods other than breast milk) [19]. If practised routinely, these practices and care behaviors could reduce newborn deaths by up to 30% [19]. It is therefore vital to measure their coverage in valid and comparable ways across countries. Unfortunately, with the exception of breastfeeding, national surveys do not routinely include information on these care behaviors. Moreover, although some South Asian countries (e.g., Nepal, Bangladesh, and India) have incorporated some of these indicators into their DHS surveys, the questions and response categories vary and are not comparable across countries. For example, in India, women are asked using a prompted question if the baby was immediately wiped dry and then wrapped, while in Nepal, women are asked separate questions about how long after birth the baby was dried and then how long after birth the baby was wrapped. The TWG has not addressed indicators for immediate behaviors and practices relevant to the mother and her care, and would encourage other groups to address this important issue.

Similar to measurement concerns around postnatal care contacts, there are methodological issues with the measurement of immediate care behaviors and practices for newborns that relate to the recall and timing of specific actions. To tackle these methodological issues, the TWG developed a list of recommended indicators, including drying/wrapping the baby, delayed bathing, and cord care. Qualitative work to assess these indicators and address measurement issues was conducted in Bangladesh and Malawi (Box 1), and different questions were tested within surveys undertaken by Save the Children in 2010 and 2011.

### Measurement Challenges and Recommendations

The qualitative research (Box 1) indicates that women can recall the event sequence for delivery and immediate care practices for newborns, that there is no difference in recall between women with facility-based births and home births, and that the timing of the birth relative to the survey has no effect on recall. Although women did have difficulty recalling the exact timing of events as measured in hours and minutes, women with facility births could estimate the amount of time elapsed between delivery and immediate newborn care. This finding is replicated in other studies [20],[21]. We recommend, therefore, that questions about care behaviors and practices for newborns should be asked for all live births, but that questions on timing should be simplified by limiting response categories to hours after birth (as opposed to minutes).

**Figure 3:**
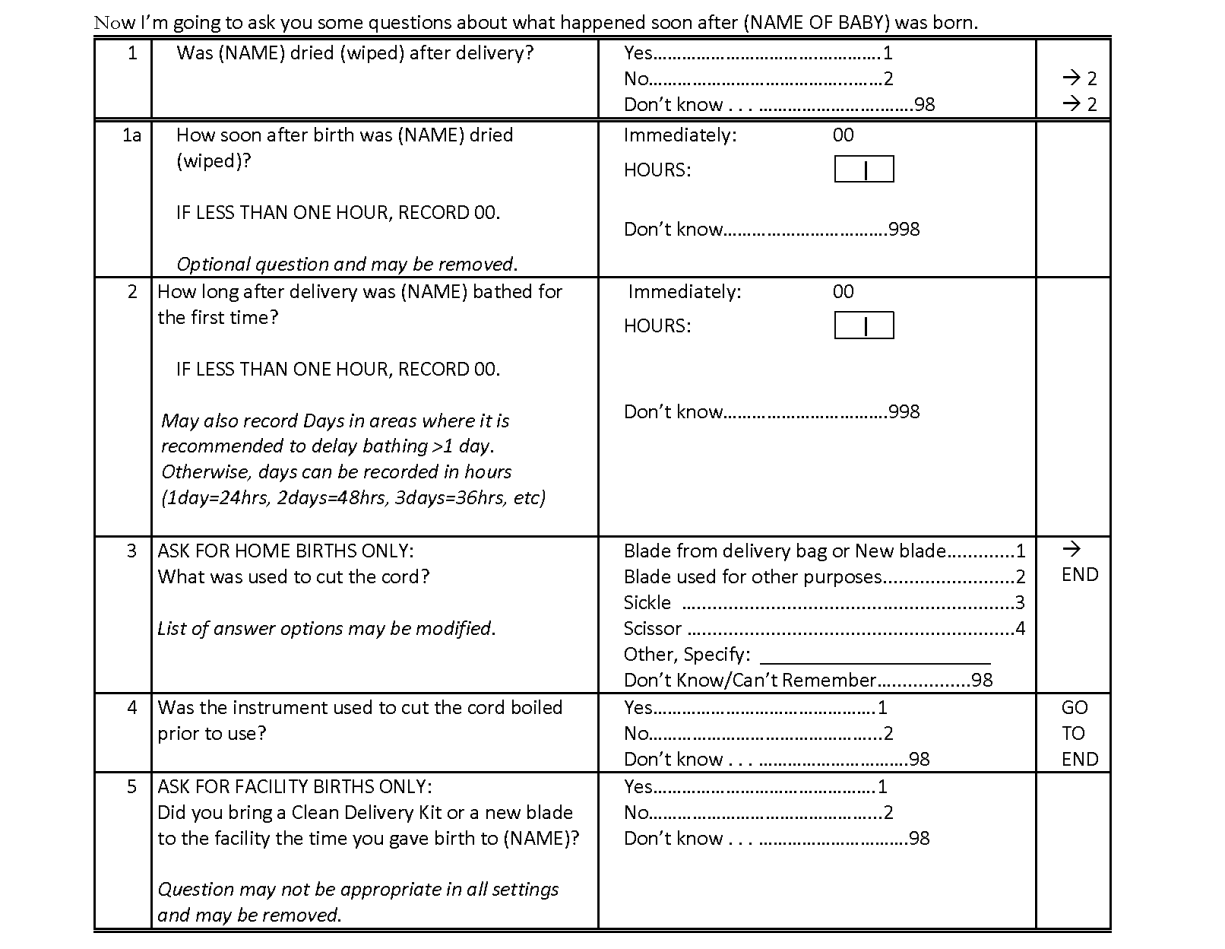
Standard questionnaire for measuring coverage of immediate newborn care.

Importantly, there was overlap between the measurement of coverage based on the indicators of drying and of wrapping the baby. Based on Save the Children survey data (Box 2), more than 90% of babies who were dried were also wrapped. Thus, it is not necessary to include indicators for both of these actions (data not shown). Survey data were also examined to assess rates of “don't know" and missing responses. Questions around delayed bathing, immediate drying, and cutting the cord for home births had low levels of “don't know" and missing responses (<10%) (Box 2). Women were less able to report on cord cutting for facility births, with “don't know" responses at higher levels. Based on these findings, we recommend three indicators for inclusion in household surveys: (1) percent of newborns dried after birth, (2) percent of newborns with bath delayed at least six hours after birth, and (3) percent of newborns with cord cut with clean instrument (for home births only) (Table 3; Figure 3). These indicators can be collected via specialized surveys, and the TWG is working with DHS and MICS on an optional newborn module. This module could also include questions on postnatal care beyond the first contact, as well as the content of postnatal care. As more data become available, these indicators may need additional revisions.

**Table 3:** Recommended indicators for care behaviors and practices for newborns.

Program Element	Indicator	Numerator	Denominator	Comments	Recommended Question(s)
**Recommended**					
Thermal care: drying	Percent of newborns dried after birth	Number of newborns dried after birth	Number of live last births in the *X* years prior to the survey	All births; timing assessment optional	Was (NAME) dried (wiped) after delivery?
Thermal care: delayed bath	Percent of newborns with first bath delayed at least six hours after birth	Number of newborns with first bath delayed at least six hours after birth	Number of live last births in the *X* years prior to the survey	All births; different timing categories can be calculated	How long after delivery was (NAME) bathed for the first time?
Cord care: clean cord cutting	Percent of newborns with cord cut with clean instrument	Number of newborns with cord cut using new blade or boiled instrument	Number of live last births (at home) in the *X* years prior to the survey	Home births only; questions on use of clean delivery kits can be included	What was used to cut the cord? Was the instrument used to cut the cord boiled prior to use?
**Additional testing needed**					
Thermal care: skin-to-skin	Percent of newborns placed on the mother's bare chest after delivery	Number of newborns placed on the mother's bare chest after delivery	Number of live last births in the *X* years prior to the survey	All births; timing assessment optional	After the birth, was (NAME) put directly on the bare skin of your chest? (Show mother example of skin-to-skin position)
Cord care: applications to the umbilical cord	Percent of newborns with nothing (harmful) applied to cord	Number of newborns with nothing (harmful) applied to cord	Number of live last births in the *X* years prior to the survey	All births; “harmful" to be defined locally	Was anything applied to the cord after the cord was cut and tied, until the cord fell off? (If “yes") What was applied to the cord?

Surveys will vary in period of recall. Typically, DHS surveys use a recall period of five years, while MICS surveys use a two-year period. Interviewer records all substances put on the cord from cutting until it falls off. Harmful substances are determined locally and split out during analysis.

### Remaining Research Gaps

Skin-to-skin care is an important intervention to maintain thermal regulation and encourage breastfeeding and bonding, but more work is needed to assess the validity of this indicator in terms of women's comprehension of survey questions concerning the intervention, as well as recall. This indicator has been recently tested in a validation study in Mozambique that is described elsewhere in this Collection [22] and is being investigated in several Saving Newborn Lives surveys. Saving Newborn Lives has also assessed a variety of indicators to look at applications to the umbilical cord after birth. The current WHO recommendation is dry cord care except in settings where the risk of bacterial infection is high [23]. However, recent evidence has demonstrated significant reductions in mortality (up to 23%) among babies who have 4% chlorhexidine applied to the cord after birth [24]–[26]. Once a global recommendation is finalized, this indicator will require further testing.

## The Way Forward for Newborn Care Indicators

Over the past several years, consistency in the measurement of newborn care interventions has improved. National data on first postnatal care contact for all newborns regardless of place of delivery are now available in four countries, and more should be forthcoming through new surveys. Development of a supplemental module is underway for national surveys to measure immediate care behaviors and practices for newborns and to provide more detailed information on postnatal care content and quality, and for visits beyond the first contact. The TWG provides a valuable forum for discussion around critical indicators using data, experience, and expert opinions.

In the future, the TWG will investigate the measurement of other evidence-based interventions that address the three main killers of newborns—complications of preterm birth, infection, and intrapartum-related deaths due to asphyxia—for which we lack coverage data. For example, facility-based Kangaroo Mother Care (care for preterm or low birth weight infants in which the baby is carried, usually by the mother, with skin-to-skin contact, and in which breastfeeding support is provided) can prevent up to half of neonatal deaths in stable newborns weighing less than 2,000 g [27]. Coverage with this intervention is not currently captured through nationally representative surveys or routine information systems but should be measurable through maternal recall. Indeed, a list of facility-based indicators to capture training of providers and coverage of Kangaroo Mother Care has been agreed upon [28], but these indicators need to be tested, refined, and incorporated into national surveys or project-based surveys. Over 700,000 newborns die of severe infection (mainly sepsis and pneumonia) each year. DHS and MICS surveys capture care seeking and treatment for fever and symptoms of acute respiratory infection for all children under five years. However, the available sample is often too small for disaggregation, and the data are not specifically presented for newborns. In addition, the symptoms assessed include only cough and difficulty breathing, so newborns with additional danger signs that indicate possible severe infection may be missed. Notably, as discussed elsewhere in this Collection, the reliability of the household-survey-based indicator that measures the proportion of children treated for pneumonia is questionable [29], which raises concerns about measuring management of other newborn problems through household surveys. Thus, innovative methodologies to complement survey research need to be developed and reviewed to improve coverage for interventions designed to manage severe infection. It is also essential that additional care provided to preterm babies and resuscitation of asphyxiated babies is tracked. The validity of asking questions about resuscitation in household surveys needs to be assessed. Facility-level data on this intervention from programs such as Helping Babies Breathe should be available soon [30].

Other indicators for maternal and newborn care require additional evaluation and development, especially for quality of care. As the percentage of facility births increases, it will become more and more critical to assess effectiveness and efficiency. Twelve million more women gave birth with a skilled attendant in 2010 compared to 2000; in South Asia, 49% of births are now assisted by skilled personnel, compared to just 30% a decade ago [4]. In this context, understanding what happens before women leave the health facility after giving birth and then at home in the postnatal period is crucial. The MICS4 module addresses these different areas of care, and different methodological approaches should be considered and differences validated [15],[16].

The TWG also advocates for improving vital registration (official registration of all births and deaths in a population), which can be linked to facility care at birth and to postnatal care [31]. Measurement of stillbirths, especially intrapartum stillbirths, and of preterm birth and low birth weight should be closely linked to improved measurement of neonatal deaths and to tracking pregnancy outcomes more comprehensively [32]. Notably, the recently published Global Burden of Disease Study 2010 did not count stillbirths, an omission that makes comprehensive tracking of global pregnancy outcomes more difficult [33]. In the future, the TWG plans to improve existing methods to capture stillbirths, by comparing data from pregnancy history modules with that from demographic surveillance sites, and by investigating the validity of shorter pregnancy history modules. The TWG will also work with partners to explore innovative methods to register births, such as using mobile phone technology. Finally, although to date the TWG has focused on measurement of newborn care and coverage through large-scale, nationally representative household surveys, in the future, it will focus more on routine data collection systems, such as health management information systems, to improve the quality of data and its use for decision-making.

## Conclusions

The world's estimated 287,000 maternal deaths, 3 million newborn deaths and 2.6 million third trimester stillbirths each year represent a huge burden that affects both families and communities. Recent increases in global attention and resource allocation for postnatal maternal and newborn care require concomitant increases in the availability of programmatic indicators and data to track change over time [34]. Progress has been made over the last five years in measurement of coverage for postnatal care and for immediate care behaviors and practices for newborns, and the TWG has provided a forum for standardizing indicators and developing common tools. However, considerable work is still needed to develop and use metrics to track progress toward improving newborn survival and care.

## Abbreviations

DHS: Demographic and Health Surveys
MICS: Multiple Indicator Cluster Surveys
TWG: Newborn Indicators Technical Working Group
UNICEF: United Nations Children's Fund
WHO: World Health Organization
